# Supplementary material for: Strain-specific evolution and host-specific regulation of transposable elements in the model plant symbiont Rhizophagus irregularis
Source: G3 (Bethesda). 2024 Mar 20;14(5):jkae055. doi: 10.1093/g3journal/jkae055 (PMC11075540; doi:10.1093/g3journal/jkae055)
Supplement: jkae055_Supplementary_Data [file jkae055_supplementary_data.zip › Figure_S1_G3-2024-404933.pdf]

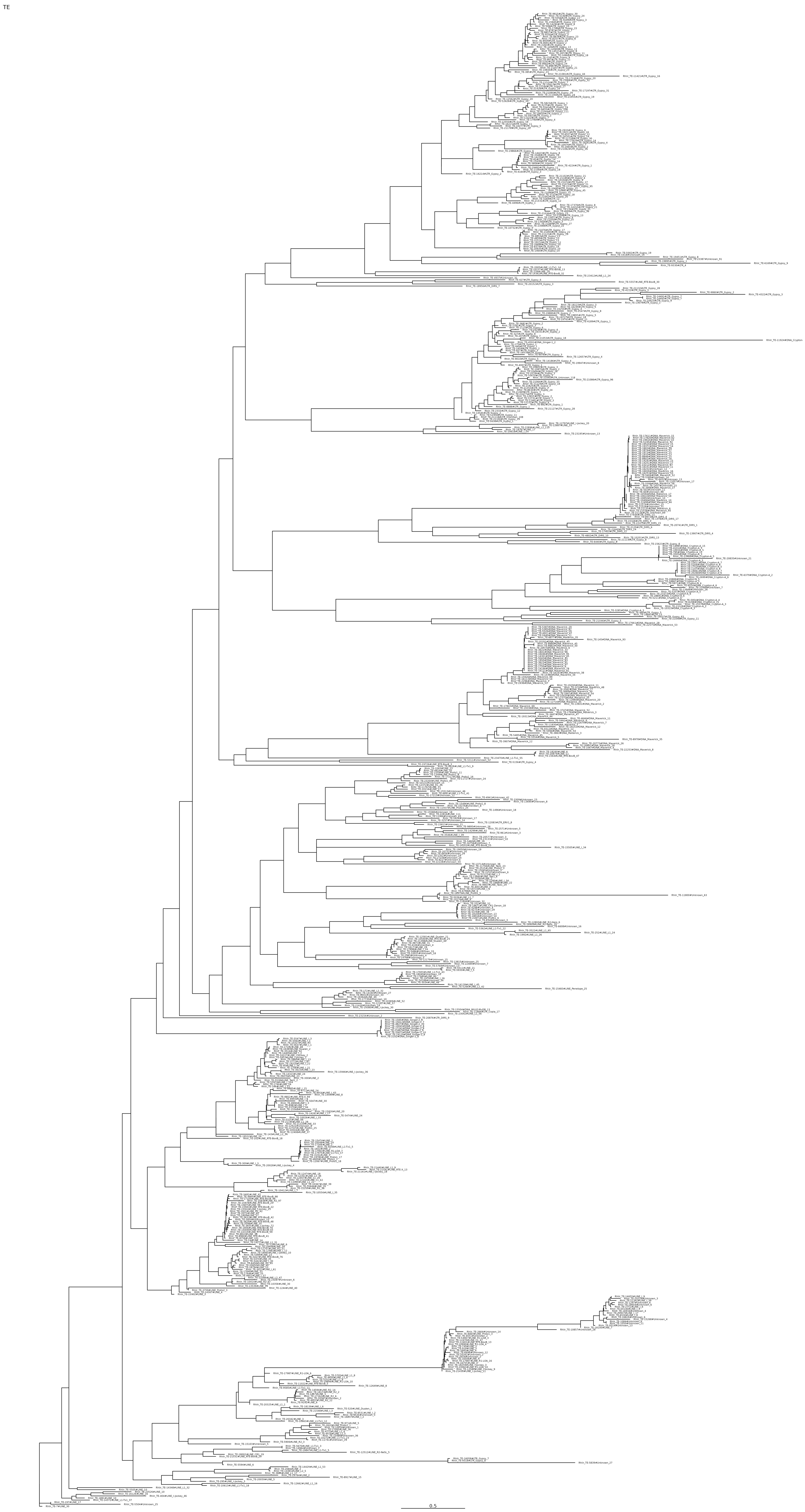

Supplementary figure S1 - Phylogenetic tree of TE from different orders with similar domains, [access it in high quality]
