# Supplementary material for: Strain-specific evolution and host-specific regulation of transposable elements in the model plant symbiont Rhizophagus irregularis
Source: G3 (Bethesda). 2024 Mar 20;14(5):jkae055. doi: 10.1093/g3journal/jkae055 (PMC11075540; doi:10.1093/g3journal/jkae055)
Supplement: jkae055_Supplementary_Data [file jkae055_supplementary_data.zip › Supplementary_Material_Legends_G3-2024-404933.docx]

**Supporting information**

**Table S1 –** Chromosome level assemblies of *R. irregularis* strains used in this study.

**Table S2 –** Domains used to construct the hmm models.

**Table S3 –** RNAseq data and alignments stats used in this study.

**Table S4 –** Number of upregulated TEs in each condition.

**Figure S1 –** Phylogenetic tree of TEs from different orders with similar domains. The figure in high resolution can be downloaded here <https://github.com/jordana-olive/TE-manual-curation/blob/main/Supplementary-figure-S1.png>.
